# Energy-efficient, real-time detection of railway fastening systems from drone-based imagery using spiking neural networks

**DOI:** 10.1038/s41598-026-53237-5

**Published:** 2026-05-21

**Authors:** Sakdirat Kaewunruen, Zibo Chen, Aryadhatu Dhaniswara, Zahid Hamarat

**Affiliations:** 1 Birmingham Center for Railway Research and Education, School of Engineering, University of Birmingham, Birmingham, B152TT UK; 2 Department of Mechanical Engineering, Boğaziçi University, Bebek, 34342 Istanbul, Turkey

**Keywords:** Spiking neural networks, Convolutional neural networks, YOLOv8, Unmanned aerial vehicle, UAV-based inspection, Energy-efficient object detection, Railway fastener detection, Engineering, Mathematics and computing

## Abstract

Rail fastener defects threaten track integrity and operational safety, making reliable automated inspection essential. This study develops an energy-efficient real-time railway fastener detection framework for UAV-based monitoring by integrating Spiking Neural Networks (SNN) into a modified Spiking-YOLOv8 architecture. Unlike conventional CNNs, the event-driven SNN processes only informative visual changes, reducing redundant computation, and achieving a better accuracy–energy balance for on-board inspection. A dataset of 173 images of ballasted railway tracks captured by UAVs was used for data augmentation, resulting in a total of 2,061 training images. The dataset is used to compare SNN and CNN performance under identical conditions, measuring accuracy, latency, and energy per inference. Results show that SNN achieves near CNN accuracy (mAP@0.5 = 0.975 vs. 0.995) while reducing energy consumption by about 80% (0.50 J vs. 2.50 J) and maintaining real-time inference speed (~ 34ms per frame). The approach demonstrates a 4.9 times improvement in accuracy-per-joule, supporting longer UAV endurance for inspection and more autonomous railway inspections.

## Introduction

**Fig. 1:**
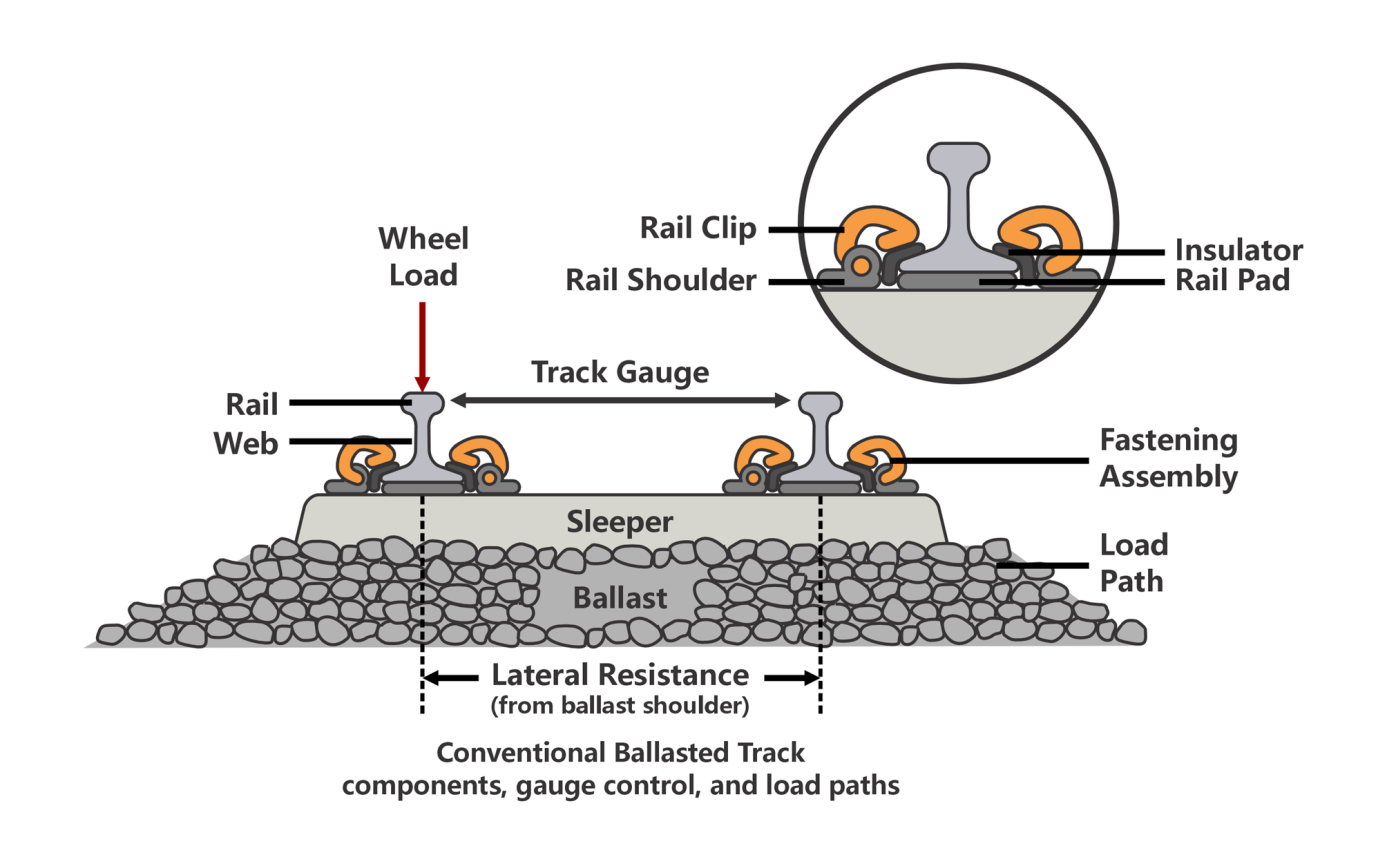
Conventional railway tracks main components (reproduced from^7^).

 A conventional ballasted track generally consists of rails, sleepers, and ballast, in which the rail and the track are connected using the fastening system as shown in Fig. 1^1–3^. The fastening system, acting together with the sleeper, maintains track gauge and rail cant, provides longitudinal and lateral restraint, electrical insulation, and mediates the transfer of wheel loads from the rail to the substructure^4,5^. Because these functions preserve track geometry and structural integrity, the condition of fastenings is safety-critical in routine inspection and maintenance^5,6^ (Fig. 1).

**Fig. 2:**
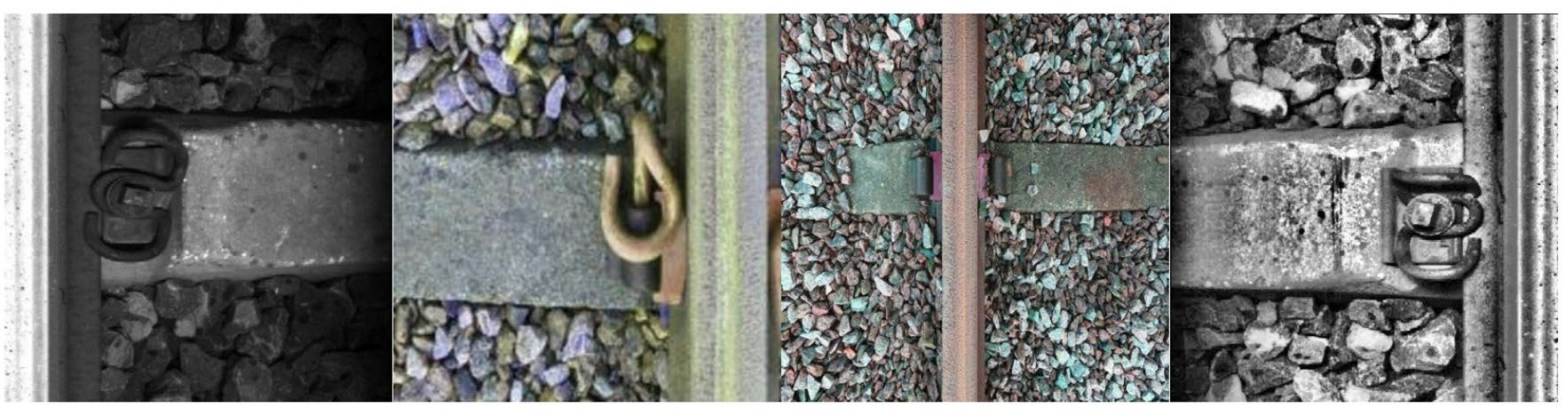
Typical rail fastening assembly and damage mechanisms (Images of ballasted railway tracks captured by UAVs dataset).

Fastener deterioration and degradation are indicated by clip fatigue/cracking or corrosion, pad crushing/creep, insulator wear/chalking with loss of insulation, cracking/spalling at concrete shoulders, and loosening of legacy bolts/anchors^8–11^. Typical rail fastening damage mechanisms shown on Fig. 2. These conditions lead to an immediate reduction in clamping force (toe load).

Fastener degradation is caused by repeated wheel–rail dynamics (impacts, heavy-axle loading, braking/traction), environmental exposure (water ingress, freeze–thaw, UV/ozone ageing), and support deficiencies (ballast fouling/settlement causing pad over-compression)^9,10,12^. Near-term consequences are reduced toe load, rail-seat stiffness change and insulation loss, manifesting as gauge widening, local misalignment and accelerated geometry deterioration^13^.

Despite the wider use of airborne imaging, workflows often rely on foot patrols or off-board batch processing, introducing delays and possessions^9^. Moving detection on-board the Unmanned Aerial Vehicle (UAV) is attractive, but embedded models are power-hungry on battery and thermally constrained platforms, encouraging approaches that maintain decision-level accuracy while substantially reducing on-board energy consumption^14,15^. Performing object detection with Convolutional Neural Network (CNN) requires hardware that consumes substantial power, which limits the operation time and decreases utility^16^. Spiking Neural Network (SNN), compared to conventional CNN, reduce power usage significantly^17,18^.

This study evaluates a SNN-based detector for Images of railway tracks captured by UAVs using a unified protocol measuring accuracy, latency, and energy per inference^19^. Unlike conventional CNNs, the event-driven SNN processes only informative changes, reducing redundant computation and achieving a better accuracy–energy balance for on-board inspection. Using UAVs promises safe, frequent, high-resolution imaging of remote or hazardous track sections, and minimizing workforce exposure and operational disruption^15,20^. Given the strict power and thermal constraints of small drones, this research focuses on achieving decision-grade accuracy with significantly lower on-board energy consumption by integrating Spiking Neural Networks (SNN) into a modified Spiking-YOLOv8 architecture.

This paper is structured as follows. Section 2 reviews related work and identifies the limitations of current fastener-detection approaches. Section 3 outlines the research objectives and describes the overall workflow of the study. Section 4 details the proposed methodology, covering data acquisition, model design, and the temporal training strategy. Section 5 presents the experimental results and provides a comparative analysis of the CNN and SNN detectors. Finally, Sect. 6 summarises the key findings, discusses operational implications for UAV-based inspection for railway and on-board processing.

## Literature review

Railway track is a system comprising rails, fastenings, sleepers, and ballast (or a slab alternative). Within this assembly, the fastening system including clips, shoulders, pads, insulators and baseplates, is securing the rail to the sleeper, maintaining gauge and rail inclination, providing longitudinal/lateral restraint and electrical insulation, and transferring load to the formation^4,5^. The performance of fastening system is governed by recognised standards, in Europe, the EN 13,481 series specifies performance requirements for fastening systems, while the EN 13,146 series prescribes laboratory test methods for longitudinal restraint, stiffness and insulation, among others; the latter includes insulation resistance criteria and environmental durability tests that directly relate to serviceability^4,12^. In North America, AREMA Chap. 5 sets recommended practice for track components and clamping/torque requirements. These documents collectively establish why fastenings are safety-critical and which properties must be maintained in service^2^.

Early artificial neural networks (ANNs) and handcrafted image processing were feasible in controlled scenes but brittle to noise, ballast clutter and exposure shifts, limiting scale^21^. From the late 2010 s onward, one-stage CNN detectors became the de-facto standard for fastener-level detection because they balanced accuracy and throughput on server-class GPUs^22,23^. “Generic DNN” classifiers appeared more in rail/sleeper condition tasks than in fastener localisation^20,24,25^. Transformers have begun to emerge in infrastructure vision, but their compute or memory profile and data demands have slowed adoption for small fastener targets on embedded devices; ensembles are mostly used for classification rather than on-board detection^25,26^. By contrast, Spiking Neural Networks (SNNs) offer energy-efficient, event-driven computation but remain under-explored in rail applications^19^. Positions of the main AI paradigms used for track inspection and the motivates of historical shift in methods shown in Table 1.

**Table 1:** Comparison of AI methods applied in railway fastener detection.

Method	Rail	Sleeper	Fastener	Other	Notes
ANN	✔	✔	×	✔	Early, low perf.
CNN	✔	✔	✔	✔	SOTA fastener detection
DNN	✔	✔	×	✔	Not optimized for detection
SNN	✔	⚬	⚬	⚬	Energy-efficient, underexplored
Ensemble methods	×	×	×	✔	Used in classification, not detection
Transformer	×	✔	⚬	⚬	Emerging in infrastructure vision

✔ : well-established, ⚬ : partial/emerging, × : not typical.

Despite impressive CNN accuracy, a knowledge gap persists in both technological approach and evaluation practice^23^. Technically, mainstream pipelines are frame-dense: every frame trigger full stacks of convolutions and memory traffic, which misaligns with battery-and thermal-limited UAVs^15,23^. Post-hoc compression (pruning/quantisation) reduces model size but does not remove per-frame dense compute, so power draw remains high and shortens flight time. Methodologically, most studies optimise or report accuracy alone, sometimes latency on desktop-class GPUs, but rarely quantify on-board energy per inference at batch = 1 with post-processing (Non-Maximum Suppression/NMS) included the measurement that governs frames-per-charge and sortie duration^18,19^. Assumptions are also narrow: datasets under-represent illumination/exposure variation and ballast clutter; short on-board temporal windows for small, repeated objects are seldom exploited; and robustness is discussed without a reproducible protocol coupling accuracy, latency, and energy^16,17^. These omissions leave industry without evidence on accuracy-per-Joule or the cost and possession implications of detector choice.

Recent advances beyond conventional CNNs motivate adoption of an event-driven approach. Spiking neural networks (SNNs) compute primarily on informative changes (threshold crossings), thereby suppressing multiply–accumulate operations and memory traffic^17^. With surrogate-gradient back-propagation-through-time (BPTT), spike-compatible modules can be trained end-to-end for detection, while short temporal aggregation windows integrate weak but persistent cues from small fasteners without incurring frame-dense compute^27,28^. Emerging neuromorphic accelerators strengthen the long-term hardware pathway for low-power inference, and standard embedded GPUs/NPUs can already benefit from sparsity in spiking activity.

Based on these developments, the study converts YOLOv8 into a spike-compatible detector by introducing SpikeConv blocks and a spiking multi-scale detection head (SpikeSPPF). The SpikeConv block consists of a convolution layer, BNAndPad, and a Leaky Integrate-and-Fire (LIF) neuron, with weights shared across timesteps to enable efficient event-driven computation. BNAndPad is a fused batch normalisation and padding operation designed to stabilise feature activations while preserving spatial alignment, whereas the LIF neuron models temporal spiking dynamics through membrane potential decay (α) and firing threshold (θ), allowing sparse and temporally aware signal propagation. In addition, SpikeSPPF is the spiking variant of Spatial Pyramid Pooling Fast (SPPF) that expands the receptive field efficiently while maintaining temporal consistency for multi‑scale features. blocks and a spiking multi-scale detection head (SpikeSPPF). The SpikeConv block consists of a convolution layer, BNAndPad, and a Leaky Integrate-and-Fire (LIF) neuron, with weights shared across timesteps to enable efficient event-driven computation. BNAndPad is a fused batch normalisation and padding operation designed to stabilise feature activations while preserving spatial alignment, whereas the LIF neuron models temporal spiking dynamics through membrane potential decay (α) and firing threshold (θ), allowing sparse and temporally aware signal propagation. In addition, SpikeSPPF is the spiking variant of Spatial Pyramid Pooling Fast (SPPF) that expands the receptive field efficiently while maintaining temporal consistency for multi‑scale features.^29–31^. Training employs surrogate-gradient BPTT with temporal-mean aggregation over a small number of steps (T ≈ 8–16). Beyond the architecture itself, a unified deployment-oriented protocol is used that jointly reports accuracy (mAP, precision, recall), batch-1 latency including NMS, and energy per inference (average device power × per-image latency)^28^. Stress tests cover grayscale, noise, exposure shifts, motion blur and ballast clutter, and ablations vary the number of time steps and aggregation rule. By holding data, pre-processing, label assignment and NMS constant across models, the analysis isolates the compute paradigm (dense CNN versus event-driven SNN) and provides evidence-based guidance that is largely absent from the current literature^30,31^.

The expected impact is operational. Lower on-board energy at near-real-time throughput enables more frames per charge, longer sorties, fewer battery swaps, and reduced track possessions, while sustaining decision-grade fastener detection. Earlier identification of fastening degradation supports targeted maintenance and can reduce inspection and possession costs, thereby improving availability on busy corridors.

## Research objective and approach

This study aims to develop and evaluate an energy-efficient real-time framework for railway fastening system detection from UAV imagery using spiking neural networks (SNNs). The research focuses on comparing the performance of the proposed SNN model with a conventional Convolutional Neural Network (CNN) baseline under identical experimental conditions. A spike-compatible variant of the YOLOv8 architecture is designed and trained using surrogate-gradient backpropagation through time (BPTT) to enable event-driven computation on UAV platforms. A unified evaluation protocol is established to jointly measure detection accuracy, inference latency, and energy consumption, ensuring fair and reproducible comparison between models.

**Fig. 3:**
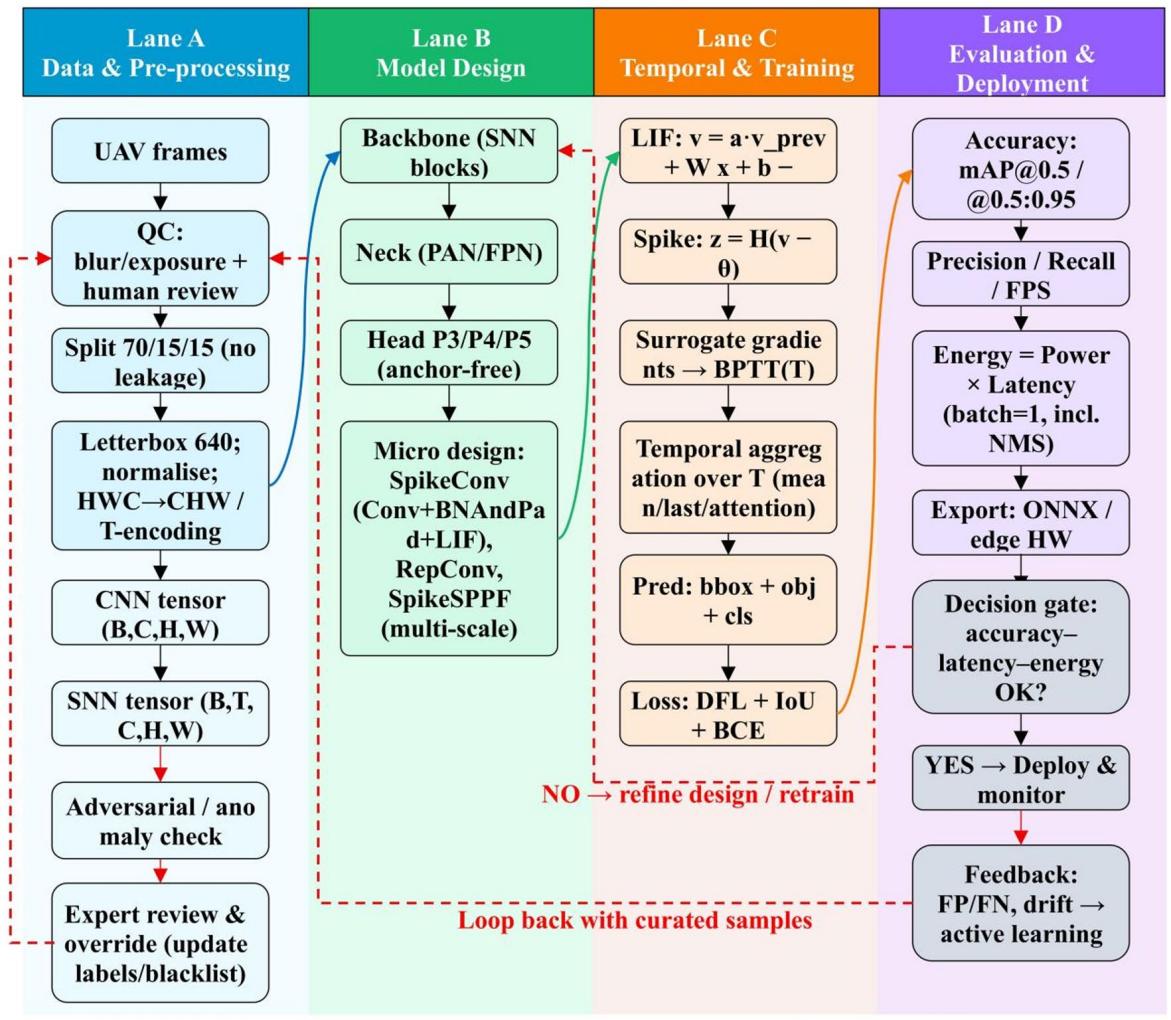
End-to-end SNN detection pipeline for innovative UAV rail inspection.

The main workflow of the study is divided into four lanes, Lanes A, B, C, and D as illustrated Fig. 3.

A: ingestion of UAV frames, automated blur or exposure checks plus human review, a leakage-free 70/15/15 split, shared minimal pre-processing, and the SNN then encodes T time steps to form a (B, T, C, H, W) tensor.

B–C: YOLO-style backbone–neck–head with spike-stable blocks and LIF dynamics; training uses surrogate-gradient backpropagation through time (BPTT) with mean temporal aggregation (variants in § 4.3).

D: evaluation reports mAP, precision/recall, Frame per Second (FPS), and energy per inference at batch size = 1, including NMS (§ 4.4); models are exported to ONNX for edge targets, and a decision gate governs deployment. The CNN baseline and the spiking model share identical data, labels, pre-/post-processing, and NMS; observed differences therefore reflect the compute paradigm.

## Methodology

**Fig. 4:**
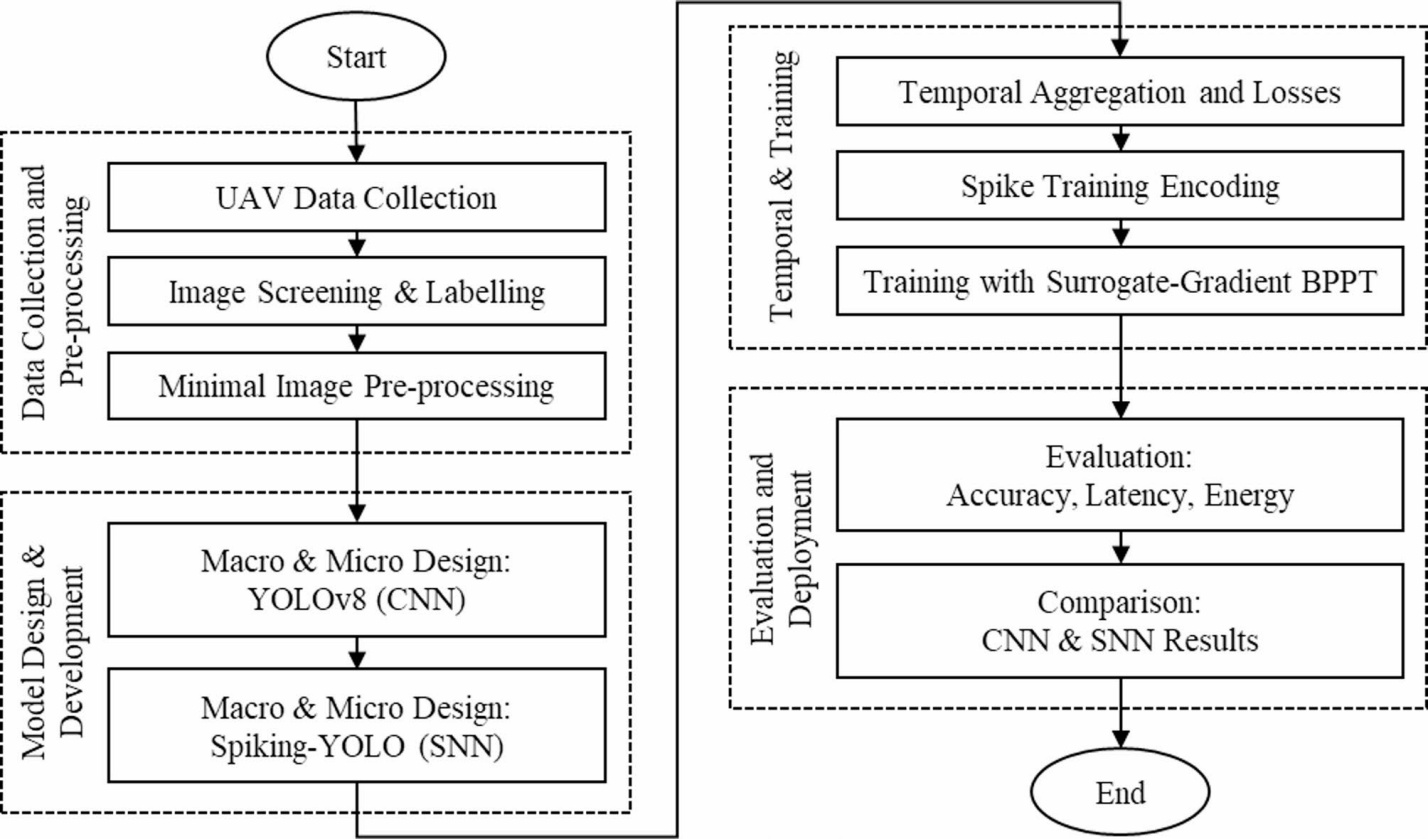
Research method flowchart.

Approach used in this study consists of four main steps, they are Data Collection and Pre-processing; Model Design and Development; Temporal and Training; and Evaluation and Deployment as shown as in Fig. 4.

### Data collection and pre-processing

This study uses Images of ballasted railway tracks captured by UAVs to automated detection of rail fastener defects, focused on missing and broken fastener. The dataset acquired using UAV through approximately 50 flight-hours covering variations in illumination (sunny or overcast, noon or dusk), alignment (tangent or curved), and visual complexity (ballast clutter, shadow, or motion blur).

Automated screening for blur screening (rejected low-sharpness images via a Laplacian-variance criterion), exposure screening (filtered extreme exposure using brightness and histogram statistics), and manual verification, a total 173 images retained including 135 RGB (78%) and 38 grayscale (22%) frame. From this dataset, data augmentation was applied, producing a total of 2,061 images for training process by transforming geometric (rotating, flipping, shearing, zooming, and shifting), transforming photometric to simulate different lighting conditions (altering brightness, contrast, saturation, or hue), and image mixing & masking (random erasing or cropping removes parts).

Labelling process comprises six classes: fastener, fastener-2 (a second fastener type), fastener_broken, fastener2_broken, missing, and trackbed_stuff. Following the YOLO text format for the annotation, one line per instance with class_id cx cy w h, where the box centre and dimensions are normalised to [0,1]. The dataset was split into training (70%), validation (15%), and testing (15%) sets by track section and acquisition time to prevent data leakage.

To ensure the fairness of the model comparison, pre-processing protocol by resizing the image into 640 × 640 pixels using letterbox scaling; convert from uint8 to float32, then scale pixel values to [0,1]; reorder channels from HWC (height–width–channels) to CHW tensors. These operations are identical for CNN and SNN experiments. This guarantees that any performance differences between CNN and SNN originate solely from the computational models rather than the data pipeline.

The imagery contains only outdoor railway infrastructure and does not include personal data. Files are anonymised using non‑identifying IDs, and metadata are retained only to support experimental reproducibility and auditability.

### Model design and development

**Fig. 5:**
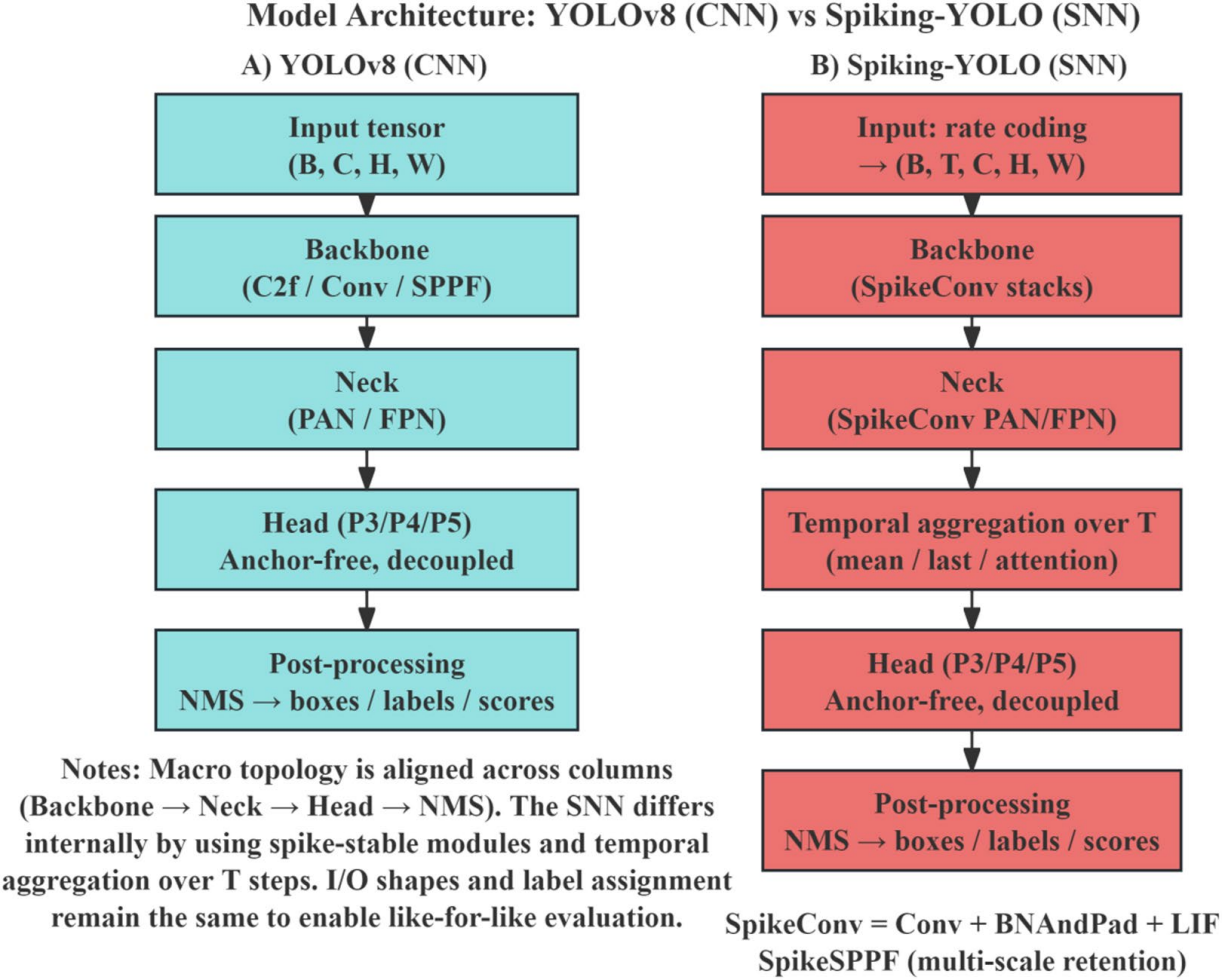
Macro comparison: YOLOv8 (CNN) vs. Spiking-YOLO (SNN).

The Spiking Detector is built on modified YOLOv8, which still using the topology of YOLOv8 to ensure a like-for-like comparison with the CNN Baseline. Input/output tensor shapes, label assignment and post-processing (including non-maximum suppression) remain identical to the YOLOv8-CNN configuration, thereby isolating the effect of the compute paradigm rather than confounding architectural changes. Macro architectures are aligned for fair comparison. YOLOv8-CNN uses Conv/RepConv and SPPF. The Spiking-YOLO preserves the backbone–neck–head topology of YOLOv8 for comparability but replaces dense activation layers with SpikeConv + LIF and SpikeSPPF, while label assignment and NMS remain identical. NMS (Non-Maximum Suppression) is a post-processing step used to remove redundant bounding boxes that overlap too much and keeps only the box with the highest confidence score for each detected object (Fig. 5).

SNNs operate in the time domain, static images must be converted into spike sequences, images are converted using MS_GetT using Bernoulli rate coding with probability p = I, where I is the normalized intensity. This process generates a temporal tensor of shape [Batch, Time, Channel, Height, Width], with T = 8–16 time-steps. The backbone and neck are built from stacked SpikeConv blocks which contain a spatial convolution whose weights are shared across timesteps, a fused BNAndPadLayer (batch-normalization plus padding), and a spiking neuron.

A spiking variant of SPPF (SpikeSPPF) is adopted to enlarge the receptive field at low computational cost while preserving temporal consistency. Multi-scale spiking features at P3, P4, and P5 are temporally aggregated (default: mean over T) and passed to a SpikeDetect head that mirrors the YOLOv8 anchor-free design, producing distributional box regression, objectness, and class probabilities.

### Temporal and training

As mentioned in § 4.2, pre-processed images are converted into temporally discrete spike sequences for spiking computation. Let Tensor $\:I\in\:[\mathrm{0,1}{]}^{B\times\:C\times\:H\times\:W}$^32^ denote the normalised batch produced in § 4.1. A binary tensor$\:\:X\in\:\{\mathrm{0,1}{\}}^{B\times\:T\times\:C\times\:H\times\:W}$ is formed by sampling$\:\:T$ discrete time steps per image via Bernoulli rate coding, with each pixel intensity serving as the spike probability. Formally, for time step $\:\mathrm{t} \in 1,\ldots, \mathrm{T}$ and channel–spatial index $\:(c,u,v)$,$\:{X}_{t}\left(c,u,v\right)\sim\:\mathrm{B}\mathrm{e}\mathrm{r}\mathrm{n}\mathrm{o}\mathrm{u}\mathrm{l}\mathrm{l}\mathrm{i}\backslash\:big\left(p=I\left(c,u,v\right)\backslash\:big\right)$^33^. Brighter locations therefore emit spikes more frequently, while darker background remains largely silent, yielding sparse event streams. To ensure a like-for-like comparison with the CNN model explained in § 4.2.

Several practical measures are adopted to stabilize training and to preserve fairness, consists of the following stages.

- I is clamped to [0,1] before sampling, an optional temperature parameter τ (default 1.0) can be used as I^τ to mildly control overall spike density.
- Encoding is performed per channel, grayscale ablations convert to a single channel prior to coding and broadcast as required by the network interface.
- Determinism is enforced by logging the random seed policy (fixed per epoch) and by recording the batch-wise mean spike density ρ, which typically lies between 0.12 and 0.22 for our dataset.
- The spike tensor is stored in [B, T, C, H, W] layout using a compact dtype (uint8 or FP16) and promoted to the compute precision at the first spiking layer to limit memory traffic.
- The CNN baseline consumes the same normalized I (without temporal replication).

Consequently, any differences in accuracy, latency, or energy per inference can be attributed to the computational model rather than to the input pathway. Alternative encodings are considered only for completeness in ablations. Poisson rate coding yields the same expectation with independent arrivals and showed negligible accuracy change relative to Bernoulli coding at higher computational cost.

**Fig. 6:**
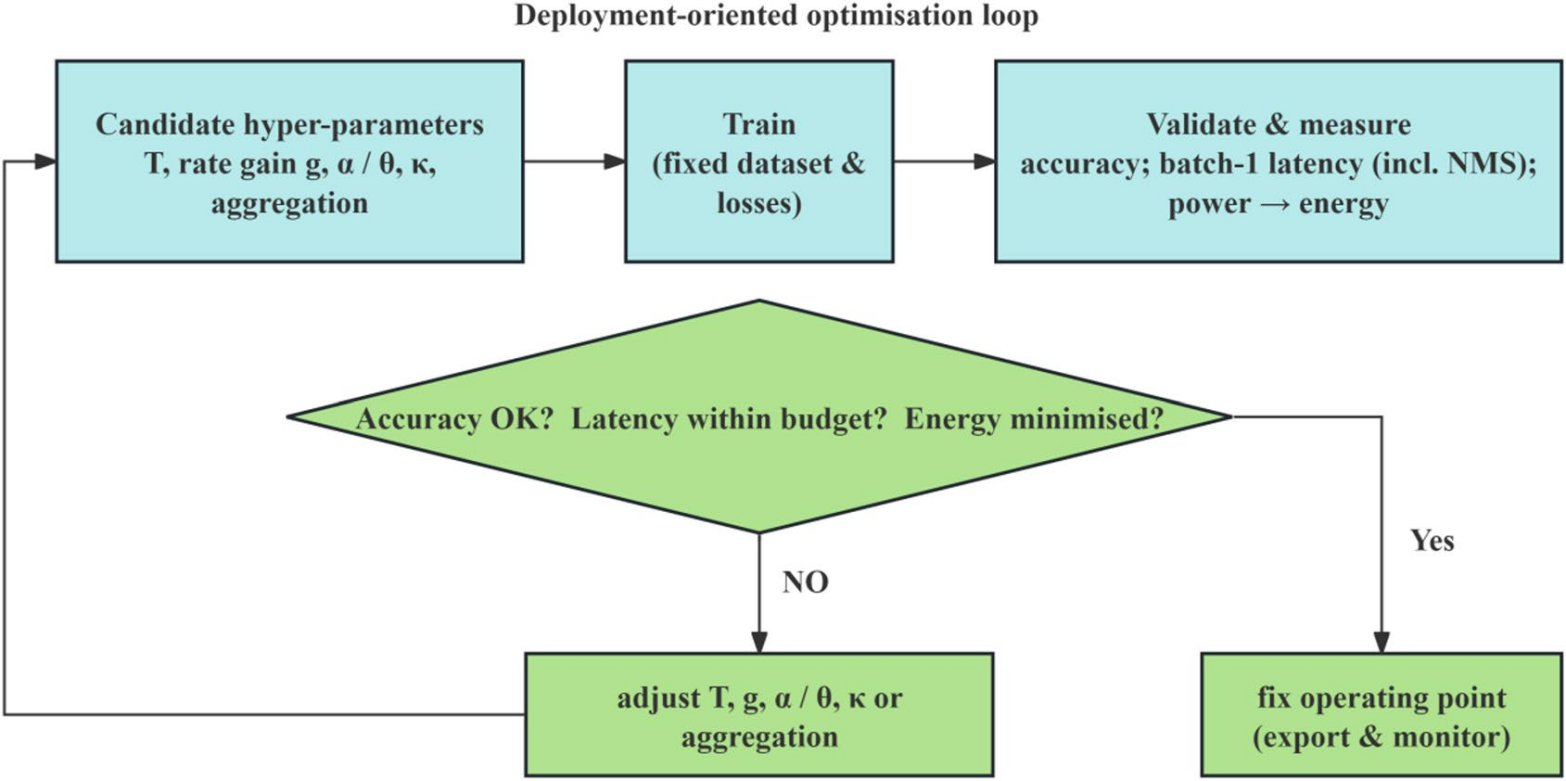
Deployment-oriented optimisation loop for the spiking detector.

**Fig. 7:**
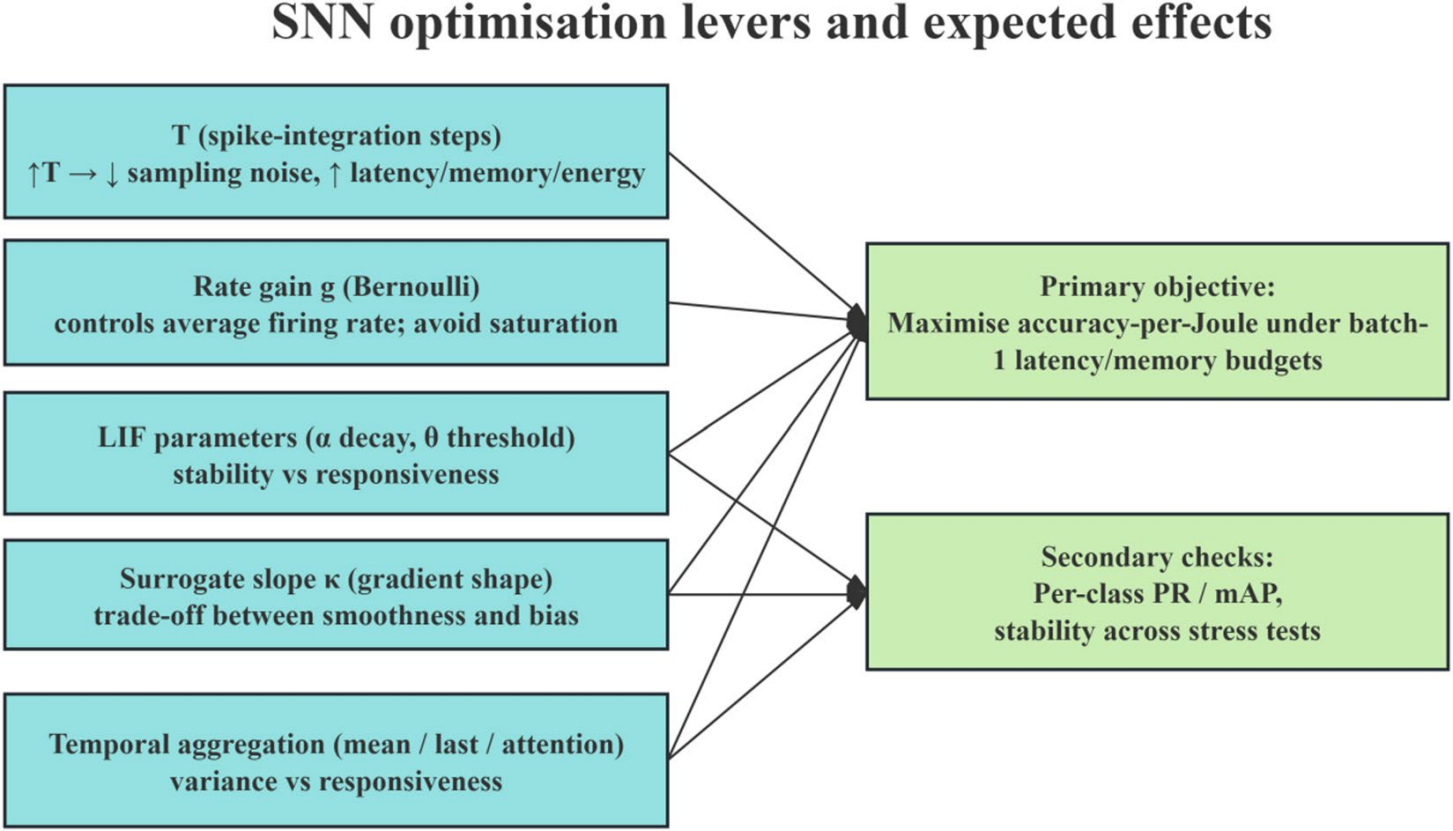
SNN optimisation levers and expected effects.

Training with surrogate gradients, stages for a deployment-oriented optimisation strategy that targets maximising accuracy-per-Joule under batch-1 latency and memory budgets. Optimisation acts only on temporal or spiking hyperparameters and on the training schedule, while data interfaces, label assignment, and post-processing remain identical to the CNN baseline, as shown in Figs. 6, 7.

**Fig. 8:**
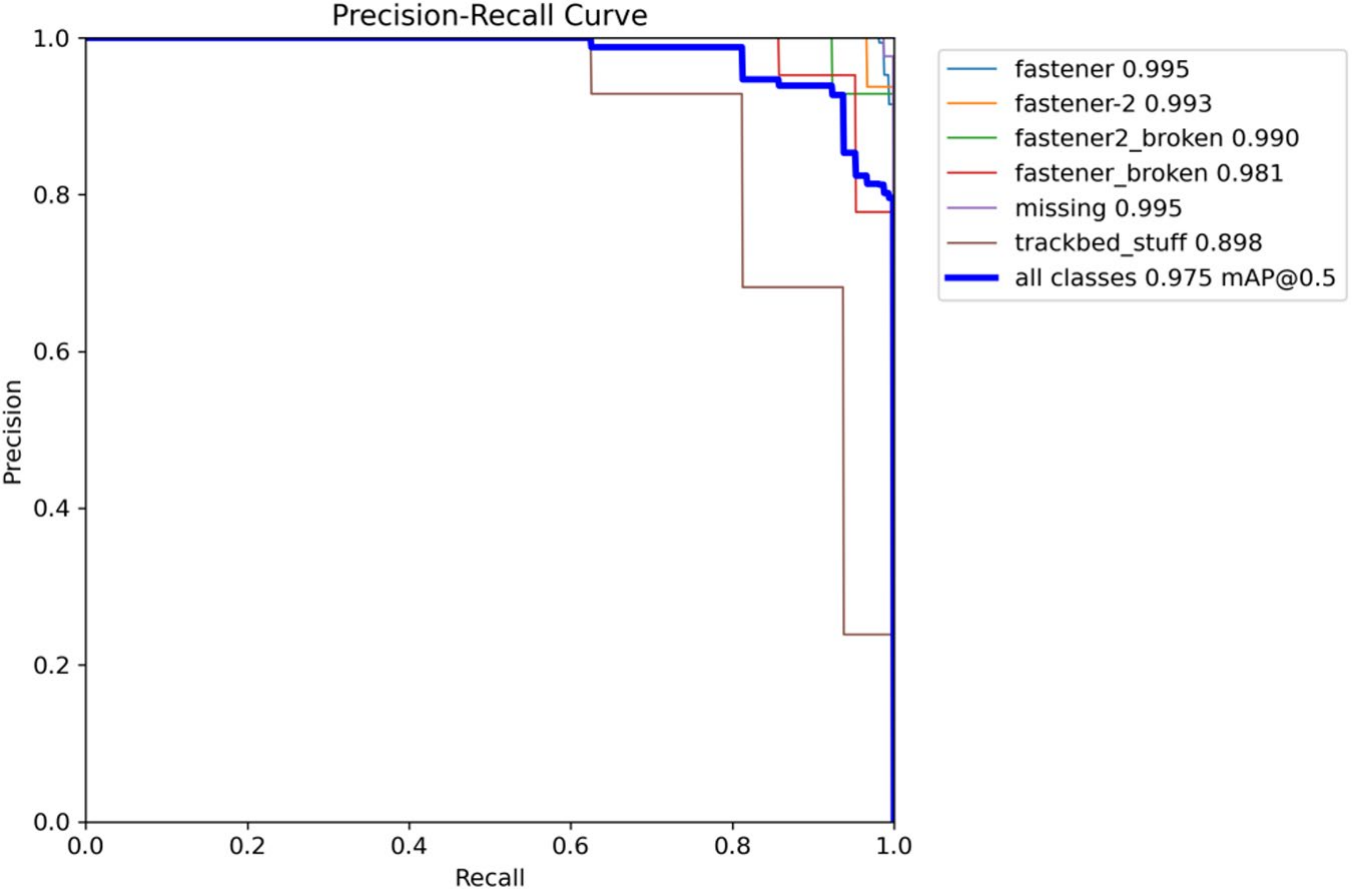
Precision–recall curves (SNN).

Model training was conducted using surrogate-gradient backpropagation through time (BPTT), which allows gradient-based learning despite the non-differentiable spike activation function. Training optimizations as shown in Fig. 8, has 5 stages included.

- Spike-integration steps T. Larger T reduces sampling noise and stabilises gradients but increases latency, BPTT activation memory, and energy approximately linearly. Unless stated otherwise, T in {8, 16} is adopted as a balanced setting.
- Rate gain g for Bernoulli coding. Normalised intensity I in [0,1] is mapped to spike probability p = clip(g*I, 0, 1). The gain controls the average firing rate; calibration avoids saturation while maintaining sufficient temporal evidence.
- LIF dynamics (alpha, theta). The decay factor alpha and the threshold theta set stability versus responsiveness; a shallow grid search identifies ranges that prevent vanishing spikes while avoiding bursty activity.
- Surrogate gradient slope kappa. The slope controls the trade-off between smoothness and bias; values that are too small give weak gradients, while overly large values increase mismatch to the step nonlinearity.
- Temporal aggregation. Aggregation is applied at the outputs of the P3, P4, and P5 feature maps for compatibility with the detection head and labels. Mean over time is the default; last-step readout and attention-weighted aggregation are examined in ablation studies.

A two-stage calibration is conducted, stage 1 performs a coarse grid over {T, g, alpha, theta, kappa} and the aggregation rule on the validation set to shortlist candidates by accuracy-per-Joule under a latency cap and stage 2 retrains the shortlisted settings to convergence and selects the operating point using a decision gate that checks accuracy, latency, and energy. All models share the same split, data pipeline, and losses, computed on temporally aggregated predictions to ensure parity with the YOLOv8-CNN baseline. Calibrating T and g reduce stochastic variance while bounding energy; tuning alpha and theta improves spike stability in deeper layers; and choosing appropriate aggregation rule balances temporal evidence against responsiveness. Collectively these adjustments improve accuracy-per-Joule at acceptable batch-1 latency without changing labels, heads, or post-processing.

### Evaluation and deployment

The proposed spiking detector is compared with a size-matched YOLOv8 baseline (v8-n or v8-s) under the same input resolution (640 × 640), pre-processing (letterbox, normalisation), and dataset split 70/15/15. The training schedule including optimiser, epochs, learning-rate policy, any deviations are stated where they occur. All models use the same split, pre-processing, label assignment, and post-processing. A fixed random seed is recorded for the split and training initialisation, and the software environment is kept consistent so that results can be reproduced. All experiments were executed on Google Colab using an NVIDIA T4 GPU (16 GB VRAM). Models were implemented in PyTorch using the Ultralytics YOLOv8 framework with Python 3.8 or higher.

Model selection is driven by validation-set Mean Average Precision (mAP), mAP@0.5 and mAP@0.5:0.95, per-class precision and recall, batch-1 latency including NMS, and energy per inference calculated as average device power multiplied by per-image latency. Inference latency is measured at batch size = 1 and includes post‑processing (NMS) to reflect deployment behaviour. Average Precision (AP) is computed as the area under the precision–recall curve obtained by sweeping the confidence threshold; mAP@0.5 is the mean of per-class AP at IoU = 0.5. IoU (Intersection over Union) measures how much the predicted bounding box overlaps with the ground truth box. The confidence threshold and NMS parameters are selected on the validation set and fixed before testing. The primary selection criterion is accuracy-per-Joule subject to latency and memory constraints consistent with on-board execution.

Energy per inference is computed as *E*_*inf*_*=P*_*avg*_ × *t*_*inf*_, where *t*_*inf*_ is the measured batch‑1 per‑image latency (including NMS) and *P*_*avg*_ is the average device power during inference. EXIF/RTK logs (timestamps and poses) are stored solely to support experiment reproducibility and to align energy measurements with inference runs. The measurement was power corresponds to GPU‑only and applied same measurement protocol to CNN and SNN to ensure a like‑for‑like comparison.

## Test results and discussion

### SNN detection results

Spiking neural network (SNN) detection results are addressed based on quantitative performance, operating threshold and error analysis, machine learning-derived insights, and qualitative analysis. Under the unified protocol, the spiking detector attains mAP@0.5 = 0.975 on the held-out test set. Per-class Average Precision (AP) values are high across safety-critical categories including, fastener 0.995, fastener-2 0.993, fastener2_broken 0.990, fastener_broken 0.981, and missing 0.995 as showed with precision-recall curve on Fig. 8. The class trackbed_stuff is used solely as hard-negative context during training and is excluded from mAP evaluation.

**Fig. 9:**
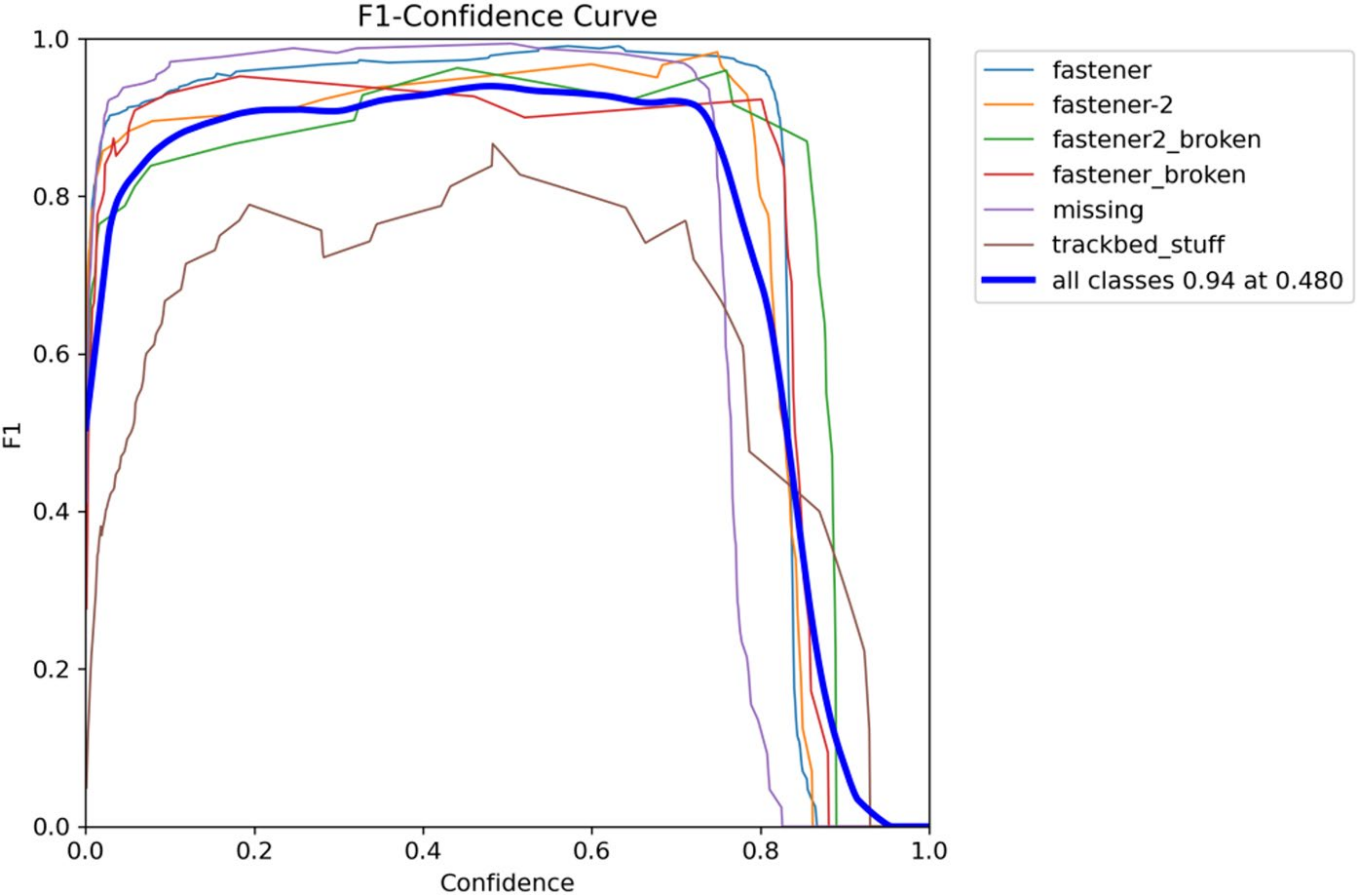
F1–confidence curve (SNN).

**Fig. 10:**
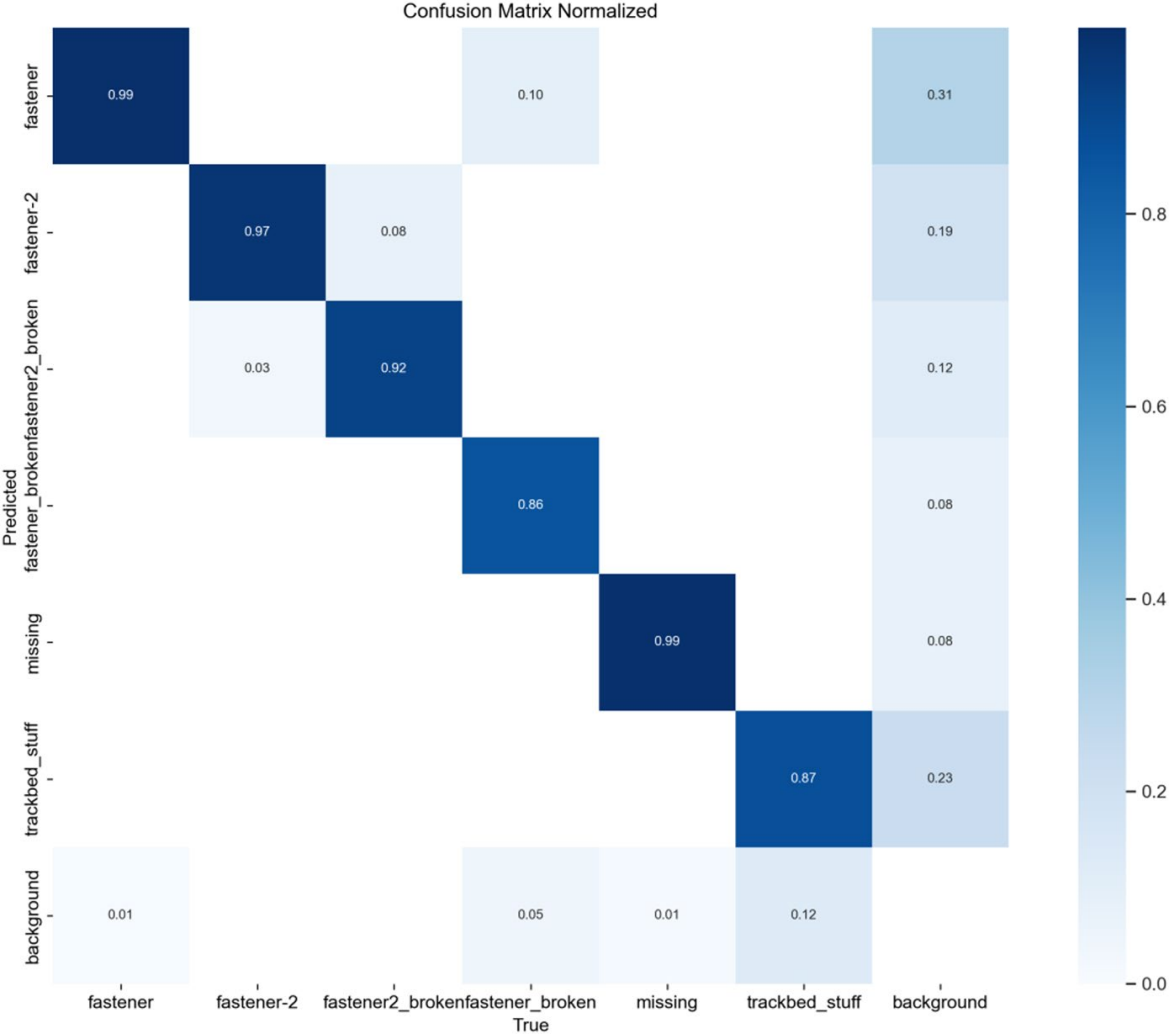
Normalised confusion matrix at τ*.

Operating threshold figured on F1-confidence curve in Fig. 9 with a clear maximum at τ* ≈ 0.48, with macro-F1 ≈ 0.94. This operating point is adopted from all threshold reports on the test set. At τ*, the normalised confusion matrix, illustrated in Fig. 10 shows strong diagonal dominance (typical diagonal entries ≥ 0.92 for the fastener classes and missing), indicating reliable discrimination among intact, broken, and missing states. Residual confusions occurred between visually similar subclasses including fastener vs. fastener-2 and the two broken variants, also in scenes affected by glare, partial occlusion, or ballast clutter.

**Fig. 11:**
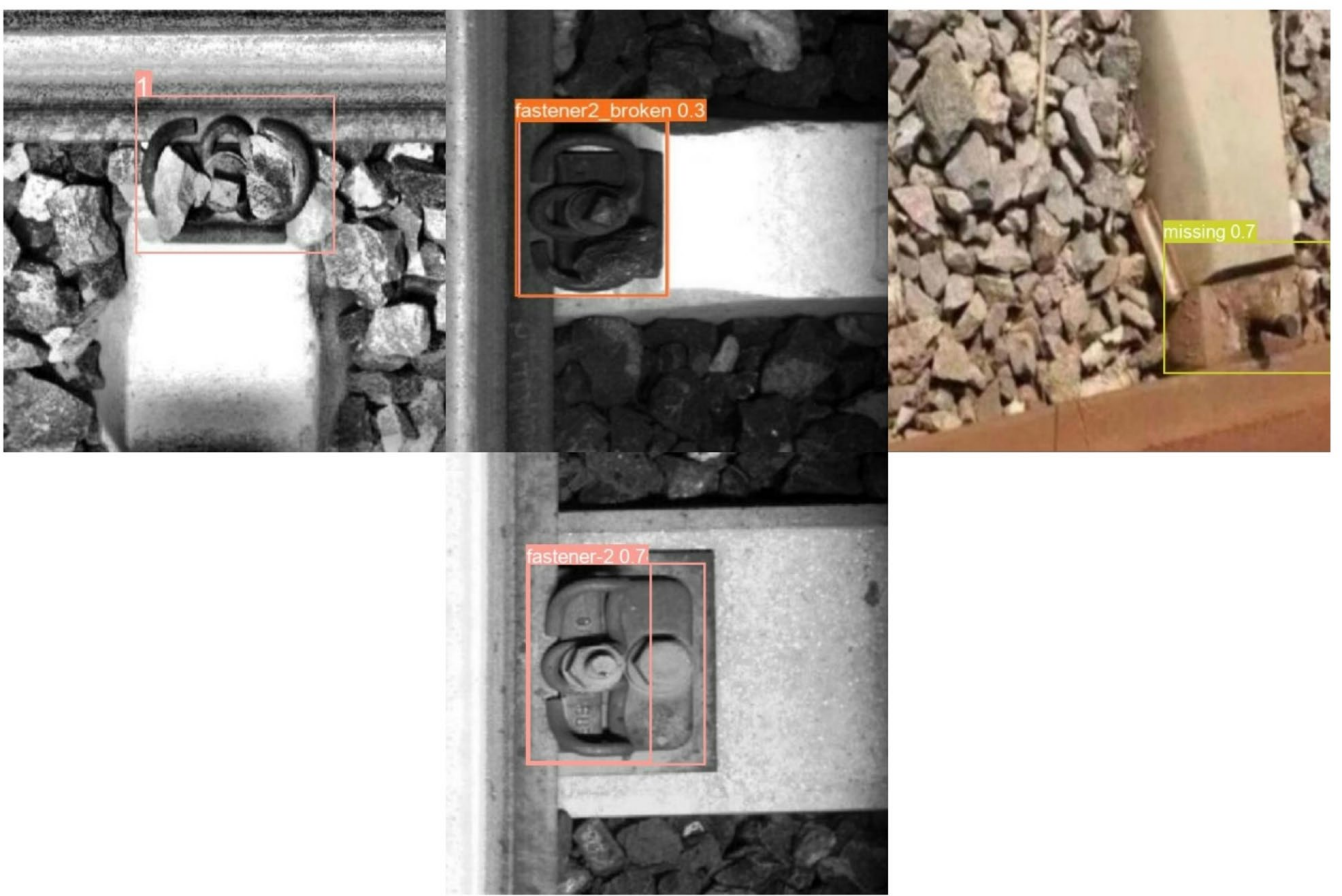
Qualitative detection.

Early model predominantly produced generic fastener boxes and struggled to separate defect under several disorder, as shown in Fig. 11. Refining and retraining the label set made the predictions became class-specific (broken and missing) and stable confidence ordering, as shown in Fig. 8, the Precision-Recall per class (AP ≥ 0.981 for all fastener categories and 0.995 for missing). The diagonal dominance in Fig. 10 indicating reliance on morphological sign (e.g., clip geometry and residual shape after breakage) rather than only on rails or sleepers.

Sample of picture overlaid under varied illumination and ballast background are shown in Fig. 11. Threshold at τ* preserves decision-grade boxes while suppressing false responses caused by glare, partial occlusion, or clutter, consistent with the quantitative trends above. The results indicate that the SNN detector achieves high mAP, shown a precise operating threshold, and yields interpretable error modes. Accordingly, the accuracy requirement for the comparative analysis in § 4.4 is satisfied.

### Main performance and the CNN–SNN trade-off

**Fig. 12:**
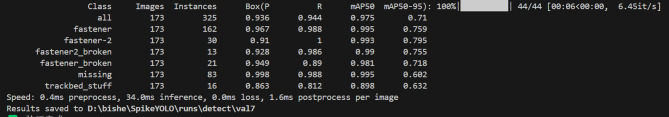
SNN validation summary.

Under the unified evaluation protocol, the SNN achieves precision = 0.936, recall = 0.944, mAP@0.5 = 0.975, and mAP@0.5:0.95 = 0.710 on the held-out test set as showed in Fig. 12. Performance based on class remains high across intact, broken, and missing fasteners. Modest dips coincide with minority classes, indicating data scarcity rather than optimisation instability. The latency decomposition, which includes pre-processing, inference, post-processing with batch size = 1 and including NMS, approximately 34ms per image.

**Fig. 13:**
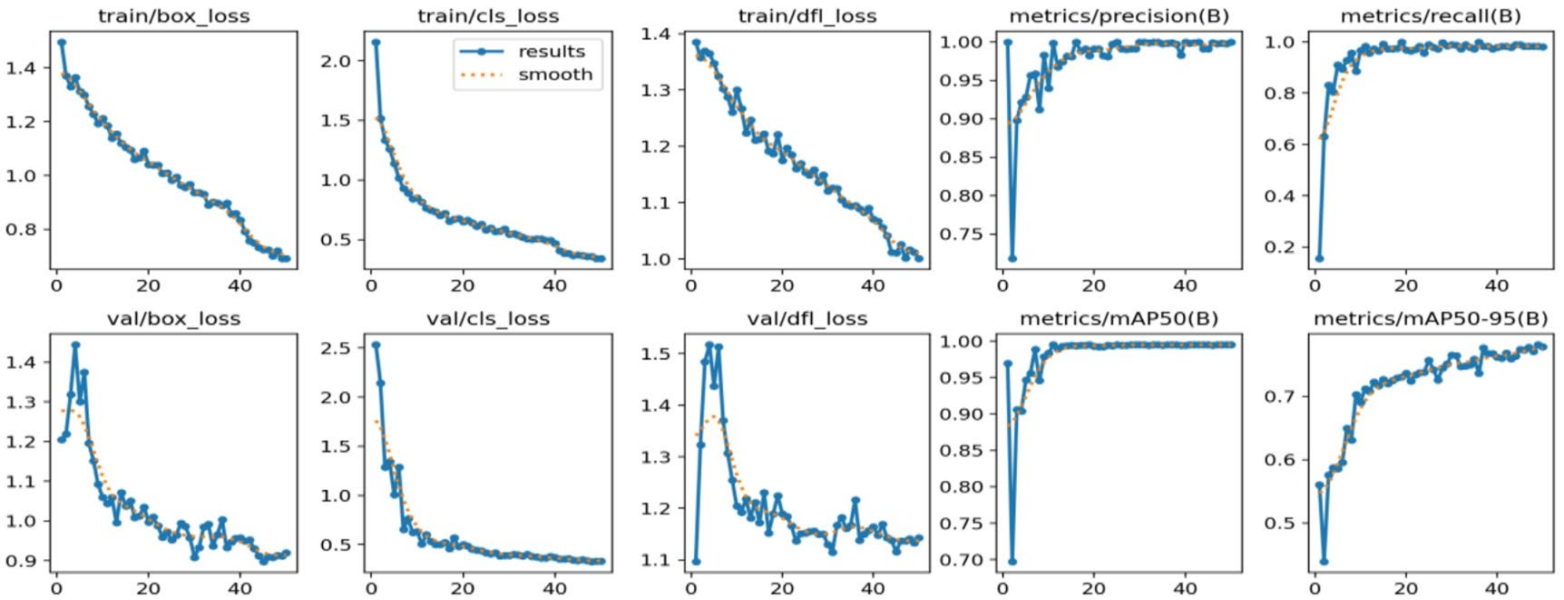
CNN (YOLOv8) validation summary.

Evaluated under same protocol, the size-matched CNN (YOLOv8) attains higher accuracy as shown in Fig. 13, with mAP@0.5 = 0.995, mAP@0.5:0.95 = 0.759, precision = 0.967, recall = 0.988, and lower per-image latency, approximately 20ms. The stricter IoU aggregate (0.5:0.95) highlights an advantage in fine localisation.

Aggregated results indicate that the CNN is both more accurate and faster than the SNN with differences ΔmAP@0.5 = + 0.020, ΔmAP@0.5:0.95 = + 0.049, and latency differences around − 14ms per image. The SNN is more energy-efficient while remaining real-time, reducing energy per inference from 2.50 J to 0.50 J, approximately 5 times or about 80% lower. The additional 14ms latency on the SNN stems from temporal unrolling over T steps, these steps allowing energy saving by event-driven sparsity (Table 2).

**Table 2:** Performance comparison CNN vs. SNN.

Metric	YOLOv8 (CNN)	Spiking-YOLO (SNN)
mAP50	0.995	0.975
mAP50-95	0.759	0.71
Precision	0.967	0.936
Recall	0.988	0.944
Inference time (ms)	20	34
Training stability	Stable	Check Val Loss
Power efficiency	High Power	Low Power
Energy per inference	2.5 J	0.5 J
Robustness	Moderate	High

Training SNNs depends on surrogate gradients, which can introduce optimisation bias and make performance highly sensitive to T, α, θ, g, and κ^26,27^. Temporal unrolling adds latency and activation memory roughly with T (observed ~ 14ms overhead vs. CNN), which can constrain small UAV processors. Energy gains rely on sparse event activity, but rate‑coded static images and limited hardware support for sparsity can moderate savings^17,18^. Tooling and neuromorphic hardware are less mature than for CNNs, complicating deployment^17,28^. A residual localisation precision gap (mAP@0.5:0.95) suggests trade‑offs between temporal aggregation and fine spatial detail.

### Strategic advantages of SNN over CNN, outlook, and study validation

**Fig. 14:**
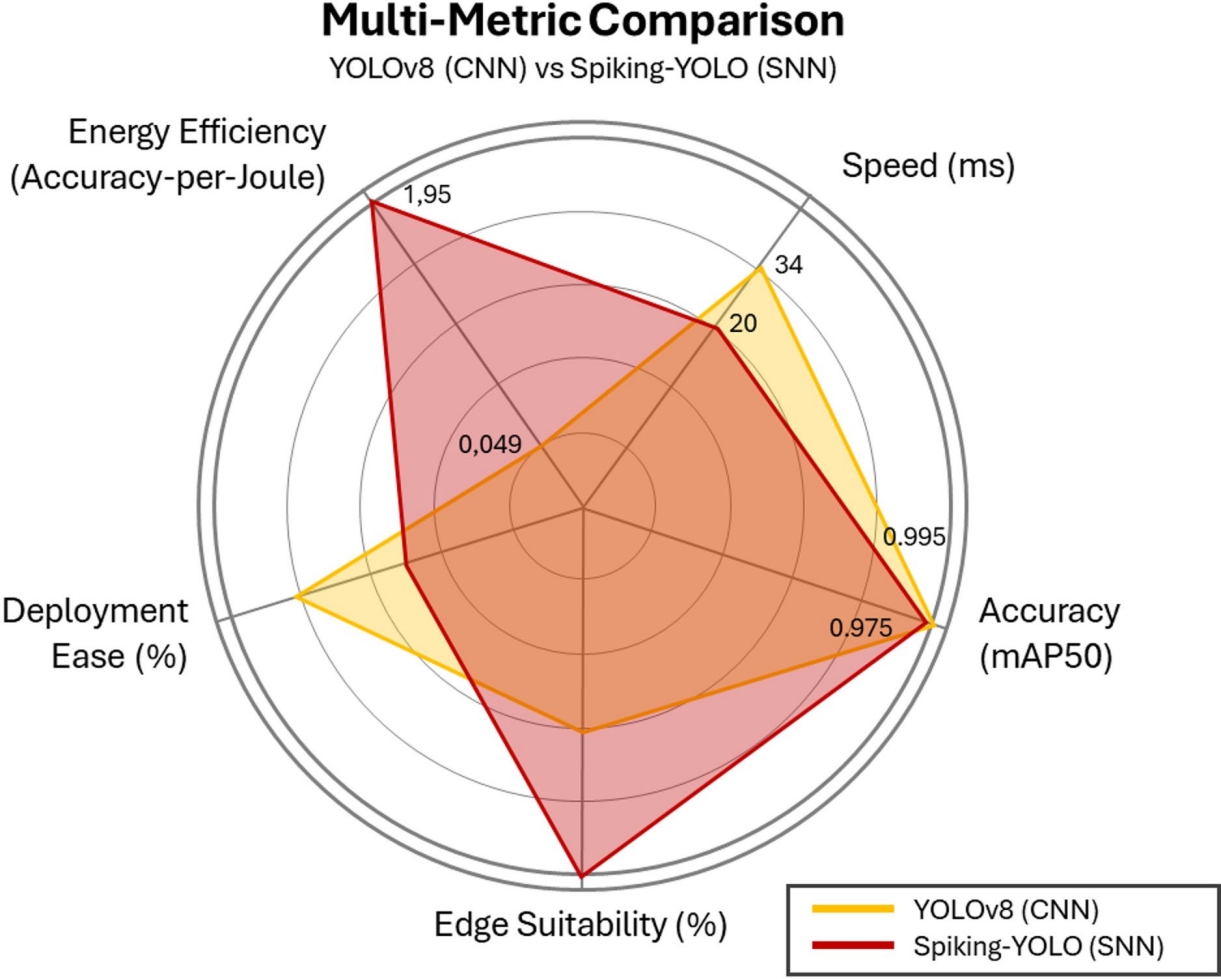
Multi–metric comparison of YOLOv8 (CNN) vs. Spiking–YOLO (SNN).

**Fig. 15:**
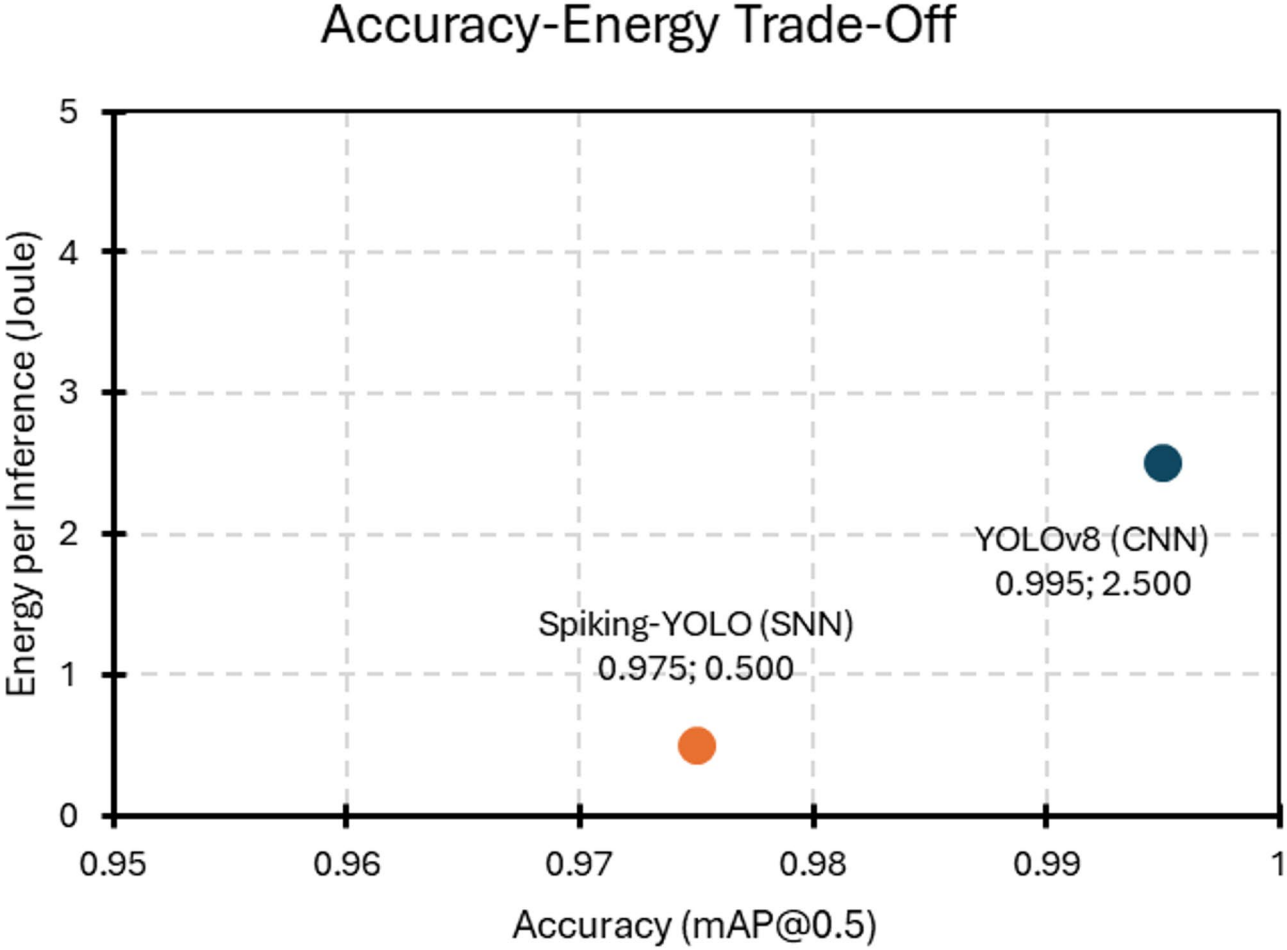
Accuracy–energy trade-off: mAP@0.5 vs. energy per inference (Joule).

Multi-metric comparison of YOLOv8 (CNN) and Spiking-YOLO (SNN) in Fig. 14 showed that the CNN is a bit higher accuracy, marginally faster, and matured ecosystem, allowing ease deployment. The SNN attains near accuracy of CNN while maintaining energy efficiency by 80%, and the SNN is best suited to edge deployment. Summarized, the SNN dominates the energy axis while remaining accuracy and real-time as showed in Fig. 15, while the CNN achieves higher accuracy and throughput corner. For railway Unmanned Aerial Vehicle (UAV) Inspection, the battery capacity is limited, making the SNN suitable, the SNN provides the superior accuracy-energy balance, whereas the CNN is preferable when higher accuracy and maximum Frame per Second (FPS) needed.

The energy efficient profile of SNN translates into longer sorties, fewer battery swaps, lower thermal load, and time for additional on-board tasks without disturbing the process. Vice versa, CNN is attractive if power is abundant and processing time must be minimised, instance high-throughput offline processing or applications demanding the strictest localisation. This study answers the core question which an SNN can provide real-time and energy-efficient fastener detection on UAV imagery without material loss of accuracy.

## Summary and conclusions

This study developed and evaluated an energy-efficient, deployment-oriented detector for UAV-based railway fastener missing and broken inspection. Using a unified protocol with identical data, pre/post-processing, batch-1 inference, and NMS across models, the spiking neural network reduced energy per inference from 2.50 J to 0.50 J (80% reduction), while maintaining near-CNN accuracy. The SNN achieved precision 0.936, recall 0.944, mAP@0.5 0.975, and mAP@0.5:0.95 0.710, while the CNN achieves higher accuracy with precision 0.967, recall 0.988, mAP@0.5 0.995, and mAP@0.5:0.95 0.759. The SNN reached real-time throughput with a median latency of 34ms and delivered a 4.90 times improvement in accuracy-per-Joule, demonstrating that the gains stemmed from the event-driven computation paradigm rather than tooling differences. These contributions collectively established the feasibility of low power and on-board detection suitable for operational UAV in railway operations.

Future research should expand the dataset especially for minority fastener classes, explore targeted augmentation, and class rebalancing to close the residual accuracy gap. Evaluating the approach across different rail types, lighting conditions, and UAV platforms would strengthen generalizability. In addition, implementing the detector on embedded GPUs/NPUs and will further benefit from neuromorphic hardware as it matures, reducing inspection cost, and improving asset availability. Integrating temporal cues from video streams, adapting the pipeline for event-based cameras, and validating performance in live operational deployments represent promising directions to advance reliability and reduce inspection costs.

## Data Availability

The data that support the findings of this study are available from the corresponding author upon reasonable request.
